# Evaluation of large language models for ICI-related liver injury with an emerging microbiome focus

**DOI:** 10.3389/fcimb.2026.1955356

**Published:** 2026-10-09

**Authors:** Man Sun, Dan Zang, Huan Zhou, Jun Chen

**Affiliations:** Department of Oncology, The Second Hospital of Dalian Medical University, Dalian, Liaoning, China

**Keywords:** gut microbiome, gut–liver axis, immune checkpoint inhibitor-related liver injury, large language models, precision medicine

## Abstract

**Background:**

Immune checkpoint inhibitor-related liver injury (ICI-LI) has heterogeneous phenotypes, a complex differential diagnosis, and evolving management. Gut microbiome research provides biological plausibility for immune regulation during ICI therapy, but evidence specific to ICI-LI remains limited and is not yet clinically actionable. We evaluated whether publicly accessible large language model (LLM) chatbots can organize this mixed evidence base and communicate its uncertainty.

**Methods:**

We developed a disease-specific benchmark of 25 questions covering immunological mechanisms, microbiome and immune regulation, diagnosis, biomarkers, and management of ICI-LI. The benchmark addressed ICI-LI broadly and included a focused microbiome component. Four publicly available LLM platforms were evaluated using DISCERN, EQIP, the Global Quality Score, the JAMA Benchmark Criteria, and six readability indices. These instruments assessed information quality, structure, transparency, and patient-facing readability rather than factual accuracy or clinical decision-making.

**Results:**

Performance differed across DISCERN, EQIP, and JAMA scores, whereas the Global Quality Score showed limited discrimination. Question-level variation was most apparent for items requiring evidence appraisal or translation. All platforms produced structured responses, but none met the prespecified patient-facing readability benchmark. Readability was interpreted separately from clinician-facing information synthesis.

**Conclusions:**

Disease-specific multidimensional evaluation is needed before LLM-generated information can be considered reliable for microbiome-informed clinical communication or decision support. Evaluation should emphasize evidence calibration and distinguish biological plausibility, association, and clinically actionable evidence.

## 1 Introduction

Immune checkpoint inhibitors (ICIs) restore antitumor immunity by blocking inhibitory pathways, including programmed cell death protein 1 (PD-1), programmed death ligand 1 (PD-L1), and cytotoxic T lymphocyte-associated antigen 4 (CTLA-4) (Ribas and Wolchok, 2018). This immune activation can disrupt self-tolerance and cause immune-related adverse events (irAEs) (Postow et al., 2018). ICI-related liver injury (ICI-LI) may require immunosuppression and interruption or permanent discontinuation of anticancer therapy (Brahmer et al., 2018; Dougan et al., 2021). Its clinical spectrum ranges from asymptomatic liver enzyme elevations to hepatitis, mixed injury, and cholangiopathy (Berry et al., 2023). Assessment therefore integrates clinical presentation, laboratory findings, imaging, treatment exposure, and competing causes.

The gut microbiome shapes systemic immunity and has been associated with both response and toxicity during ICI therapy (Gopalakrishnan et al., 2018; Matson et al., 2018; Regev et al., 2020; Andrews et al., 2021; Lee et al., 2022). Microbial composition and barrier integrity, together with microbiota-derived metabolites, can alter immune activity and host metabolism. The gut-liver axis links these processes to hepatic immune regulation: dysbiosis, barrier failure, and altered metabolites may disturb immune tolerance within the liver (Tripathi et al., 2018; Albillos et al., 2020). Yet microbiome findings vary across cancer types, treatment regimens, populations, sequencing methods, and analytical pipelines (Lee et al., 2022). Most microbiome evidence in this setting concerns ICI response or irAEs across cancer types rather than liver-specific toxicity. Reported signatures remain associations rather than validated biomarkers, and clinical interpretation must distinguish biological plausibility from evidence strong enough to guide care.

Large language model (LLM) chatbots are increasingly used by clinicians and patients to obtain biomedical information (Kung et al., 2023; Singhal et al., 2023). They can retrieve and summarize complex material within seconds. For evidence-intensive, cross-disciplinary problems such as ICI-LI, this speed may help users organize relevant knowledge. Their reliability, however, remains uncertain. Medical chatbot studies have largely examined general health education, question answering, or performance within a single specialty. Reported limitations include incomplete information, opaque sourcing, outdated knowledge, and technical language that can impair clinical interpretation and patient understanding (Ayers et al., 2023; Lee et al., 2023).

LLMs have rarely been tested on specialist questions that combine established clinical guidance with emerging microbiome evidence. The relevant task is clinical information synthesis: responses must organize evidence, state uncertainty, and avoid presenting associations as actionable biomarkers. Whether current platforms provide this calibrated synthesis remains unclear.

We therefore developed a disease-specific benchmark covering the broader clinical spectrum of ICI-LI with a focused microbiome component. We compared information quality, structure, transparency, and readability across platforms. The benchmark examines clinical information synthesis and evidence calibration, not diagnostic accuracy or clinical decision-making competence.

## 2 Methods

### 2.1 Study design and overview

This cross-sectional comparative study evaluated the quality, transparency, and readability of LLM-generated medical information about ICI-LI. We used a benchmark design with controlled inputs and a predefined collection procedure. Every question was submitted without modification, simplification, or added context. Because the study used only publicly available AI-generated text and involved no participants, patient data, biological specimens, or clinical interventions, ethics approval and informed consent were not required.

### 2.2 Development of the question dataset

We developed questions that sampled clinically relevant aspects of ICI-LI. Their content was based on current evidence for ICI-associated hepatotoxicity, microbiome-mediated immune regulation, clinical assessment, treatment, and translational research.

The final dataset comprised 25 questions (**Table 1**) across five domains: immunological mechanisms; the gut microbiome and immune regulation; clinical diagnosis and differential assessment; biomarkers and risk prediction; and therapeutic management and future applications. The benchmark therefore assessed ICI-LI broadly rather than treating the microbiome as an established clinical biomarker domain. Microbiome items were included to test how platforms handle a biologically plausible but clinically immature evidence base. All questions were specified before data collection and submitted unchanged to each chatbot.

**Table 1:** Benchmark questions on ICI-related liver injury with a focused microbiome component.

Question no.	Clinical question
1	What are the major immunological mechanisms underlying immune checkpoint inhibitor-related liver injury?
2	How does PD-1/PD-L1 or CTLA-4 blockade disrupt hepatic immune tolerance and cause liver injury?
3	What are the differences between immune checkpoint inhibitor-related hepatitis and conventional drug-induced liver injury?
4	How do different immune checkpoint inhibitors influence the incidence and mechanisms of liver toxicity?
5	Why do only some patients receiving immune checkpoint inhibitors develop liver injury despite similar treatment exposure?
6	What role does gut microbiota play in the development of immune checkpoint inhibitor-related liver injury?
7	How can gut microbiota influence systemic immune activation during immune checkpoint inhibitor therapy?
8	Which gut microbial signatures have been associated with immune checkpoint inhibitor toxicity, and what is the strength of evidence?
9	Can *Akkermansia muciniphila* be used as a biomarker for predicting immune checkpoint inhibitor-related liver injury?
10	How might microbial metabolites such as SCFAs and bile acids regulate immune checkpoint inhibitor-related liver injury?
11	A patient with advanced NSCLC develops elevated ALT and AST after PD-1 inhibitor therapy. How should clinicians evaluate this condition?
12	What differential diagnoses should be excluded before diagnosing immune checkpoint inhibitor-related hepatitis?
13	How can clinicians distinguish immune checkpoint inhibitor-related hepatitis from liver metastasis or other causes of liver dysfunction?
14	What clinical and biological biomarkers can predict the risk of immune checkpoint inhibitor-related liver injury?
15	Could gut microbiome biomarkers complement conventional indicators for early detection of immune checkpoint inhibitor-related liver injury?
16	How should immune checkpoint inhibitor therapy be managed after diagnosis of immune checkpoint inhibitor-related hepatitis?
17	When and how should immune checkpoint inhibitors be rechallenged after liver toxicity?
18	Can probiotics or prebiotics prevent immune checkpoint inhibitor-related liver injury? Discuss current evidence.
19	What is the potential role of fecal microbiota transplantation in preventing or treating immune checkpoint inhibitor-related adverse events?
20	Should microbiome-modulating interventions be recommended routinely for patients receiving immune checkpoint inhibitors?
21	What are the major limitations preventing translation of microbiome biomarkers into clinical practice for immune checkpoint inhibitor toxicity prediction?
22	How should researchers design prospective studies investigating gut microbiome biomarkers for immune checkpoint inhibitor-related liver injury?
23	What types of multi-omics approaches could improve prediction of immune checkpoint inhibitor-related liver injury?
24	How can artificial intelligence integrate microbiome, clinical, and immunological data to improve management of immune checkpoint inhibitor-related liver injury?
25	What are the future directions for personalized prevention and management of immune checkpoint inhibitor-related liver injury?

### 2.3 LLM selection and response collection

We evaluated four publicly available LLM-based chatbots: ChatGPT 5.5, Gemini 3.5 Flash, Perplexity, and Copilot. These platforms were selected because they represent widely accessible LLM-based systems with substantial user adoption and application in medical information retrieval. Each chatbot received the same questions without added prompts, context, or follow-up interactions. Responses were collected from the public web interfaces under default settings and retained as raw text. For readability analysis, we converted responses to plain text and removed symbols, bullet points, numbering, hyperlinks, citation markers, and separate reference lists without changing their wording or meaning. The evaluation was performed in July 2026 under publicly available default versions.

### 2.4 Assessment of chatbot-generated information

We assessed response quality and transparency with four established instruments: DISCERN, the Ensuring Quality Information for Patients (EQIP) tool, the Global Quality Score (GQS), and the JAMA Benchmark Criteria (Silberg et al., 1997; Charnock et al., 1999; Moult et al., 2004; Bernard et al., 2007). These instruments capture complementary features of treatment information, educational structure, perceived quality, and source transparency (**Supplementary Table 2**). Their scores were interpreted as indicators of information quality, structure, and transparency rather than direct measures of factual accuracy or clinical reasoning.

DISCERN assessed source transparency, completeness, and balance in treatment information (Charnock et al., 1999). Scores ranged from 16 to 80, with higher scores indicating better quality: excellent (63-80), good (51-62), fair (39-50), poor (27-38), or very poor (16-26).

The 20-item EQIP tool assessed the quality, structure, and usability of health education materials (Moult et al., 2004). Each Yes response received 1 point and each No response 0 points. We calculated the final score as (total score/20) × 100 and classified it as excellent (76-100), good (51-75), poor (26-50), or indicating extremely serious quality problems (0-25).

The GQS rated information organization, clarity, and usefulness on a five-point scale from 1 (very poor) to 5 (excellent) (Bernard et al., 2007). The JAMA Benchmark Criteria assessed authorship, attribution, disclosure, and currency; each criterion received 0 or 1 point, for a maximum score of 4 (Silberg et al., 1997).

Two medically trained reviewers independently rated every response (Ojha et al., 2021; Gkikas et al., 2022). Before formal assessment, they scored a random 10% sample to calibrate interpretation of the instruments. Reviewers were blinded to chatbot identity. Disagreements were discussed, and a third senior reviewer adjudicated unresolved cases.

### 2.5 Readability analysis

We measured linguistic complexity with six indices: the Automated Readability Index (ARI), Flesch Reading Ease Score (FRES), Gunning Fog Index (GFI), Flesch-Kincaid Grade Level (FKGL), Coleman-Liau Index, and Simple Measure of Gobbledygook (SMOG) (Mac et al., 2022; Özduran and Hanci, 2022; Yilmaz Hanci, 2023; Hanci et al., 2024) (**Supplementary Table 3**). Higher grade-level scores indicate more difficult text, whereas higher FRES values indicate easier reading. A sixth-grade reading level was prespecified as the reference threshold for patient-oriented information, consistent with widely cited health-literacy guidance (Berkman et al., 2011; Ojha et al., 2021; Gkikas et al., 2022). The benchmark questions were primarily clinician-facing. Readability was analyzed separately to assess whether unmodified responses would also be suitable for patient-facing communication; it was not used as a measure of specialist content quality.

### 2.6 Statistical analysis

Continuous variables were summarized as median (interquartile range), and categorical variables as counts and percentages. Differences in quality and readability measures among chatbots were tested using the Kruskal-Wallis test. Significant omnibus results were followed by Dunn’s pairwise comparisons with Holm correction. Readability scores were compared with the prespecified sixth-grade threshold using the Wilcoxon signed-rank test. Kruskal-Wallis effect sizes were reported as epsilon squared (ϵ²). Analyses were performed using Python version 3.10.4.

## 3 Results

### 3.1 Characteristics and scope of the benchmark question dataset

The benchmark comprised 25 clinically oriented questions on LLM-generated information about ICI-LI. Five questions were assigned to each of five domains.

The domains covered immunological mechanisms, microbiome-mediated immune regulation, diagnosis and differential assessment, biomarkers and risk prediction, and therapeutic management with future translational applications. Together, they sampled mechanistic interpretation, evidence appraisal, clinical assessment, treatment decisions, and precision-medicine concepts. **Supplementary Table 1** lists every question and its assigned domain.

### 3.2 Overall quality assessment reveals heterogeneous performance among LLM platforms

DISCERN, EQIP, and JAMA scores differed among the four chatbots, whereas GQS scores did not (**Table 2**). Kruskal-Wallis tests showed between-platform differences for DISCERN (P < 0.001, ϵ² = 0.212), EQIP (P < 0.001, ϵ² = 0.369), and JAMA (P < 0.001, ϵ² = 0.714). GQS scores were comparable (P = 0.826, ϵ² = 0.000).

**Table 2:** Quality scores of AI chatbots.

Program	ChatGPT	Gemini	Copilot	Perplexity	ϵ²	P
DISCERN	38.00(36.00, 42.00)	38.00(35.00, 42.00)	43.00(42.00, 47.00)	43.00(41.00, 48.00)	0.212	<0.001
EQIP	55.00(50.00, 60.00)	50.00(45.00, 60.00)	70.00(60.00, 75.00)	70.00(60.00, 70.00)	0.369	<0.001
JAMA	0.00(0.00, 0.00)	0.00(0.00, 0.00)	1.00(1.00, 1.00)	1.00(1.00, 1.00)	0.714	<0.001
GQS	4.00(4.00, 4.00)	4.00(4.00, 4.00)	4.00(4.00, 4.00)	4.00(4.00, 4.00)	0.000	0.826

Values are shown as median (Q1, Q3). P values were obtained using the Kruskal-Wallis test; ϵ² denotes the Kruskal-Wallis effect size.

Copilot and Perplexity had higher median scores in selected quality domains (**Figure 1**). Their median DISCERN scores were both 43.00, compared with 38.00 for ChatGPT and Gemini. Median EQIP scores were 70.00 for Copilot and Perplexity, 55.00 for ChatGPT, and 50.00 for Gemini. Platform differences were therefore more apparent in completeness, reliability, and educational content than in perceived global quality.

**Figure 1:**
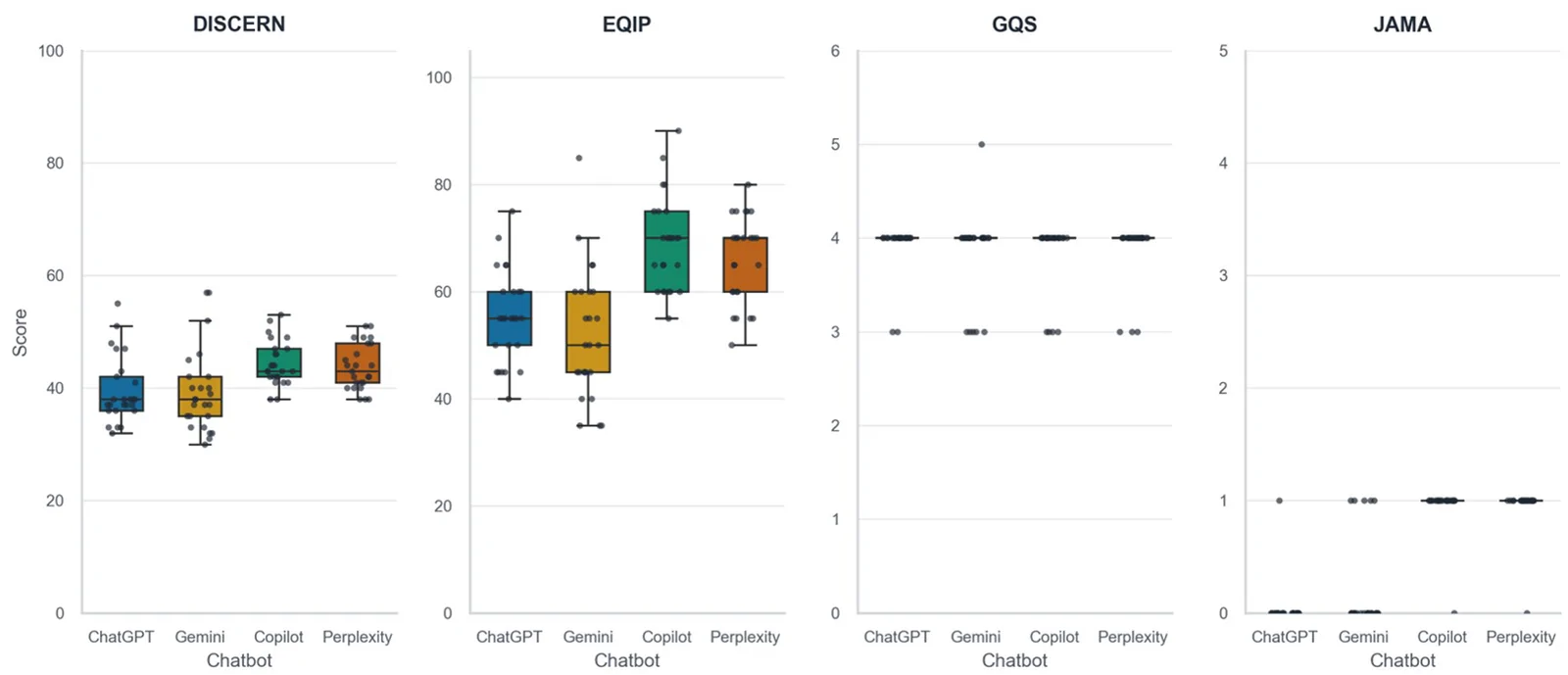
Quality of LLM-generated medical information across chatbot platforms. Boxplots show DISCERN, EQIP, Global quality score (GQS), and JAMA Benchmark Criteria scores for the four chatbots. Each point represents one question-level assessment. Higher scores indicate better treatment information, educational quality, perceived response quality, or transparency, as defined by the corresponding instrument.

Transparency produced the largest effect size. Median JAMA scores were 0 for ChatGPT and Gemini and 1 for Copilot and Perplexity (ϵ² = 0.714). By contrast, the identical median GQS scores across platforms suggest that global subjective assessments may fail to detect clinically meaningful differences identified by structured evaluation instruments.

### 3.3 Pairwise comparisons between LLM platforms

Dunn pairwise tests with Holm correction localized differences between platforms for each evaluation instrument (**Table 3**). Differences were concentrated in structured measures of information quality and transparency rather than the global impression score.

**Table 3:** Pairwise comparisons of AI chatbots (P values).

Pairwise comparison	DISCERN	EQIP	JAMA	GQS
ChatGPT-Copilot	0.002	<0.001	<0.001	1.000
ChatGPT-Gemini	1.000	1.000	1.000	1.000
ChatGPT-Perplexity	0.012	0.002	<0.001	1.000
Copilot-Gemini	0.001	<0.001	<0.001	1.000
Copilot-Perplexity	1.000	1.000	1.000	1.000
Gemini-Perplexity	0.007	<0.001	<0.001	1.000

Cells show Holm-adjusted P values from Dunn’s *post hoc* test. NA indicates that no applicable comparison result was available.

For DISCERN, ChatGPT scored lower than Copilot and Perplexity; Gemini also scored lower than Copilot and Perplexity. The platforms thus differed in the reliability, completeness, and balance of treatment information.

EQIP showed the same grouping. ChatGPT differed from Copilot and Perplexity, as did Gemini. ChatGPT and Gemini did not differ, and neither did Copilot and Perplexity.

JAMA comparisons again separated ChatGPT and Gemini from Copilot and Perplexity. Differences were found for ChatGPT versus Copilot, ChatGPT versus Perplexity, Gemini versus Copilot, and Gemini versus Perplexity, but not within either pair. No GQS comparison was significant.

No platform was consistently superior across all instruments; performance depended on the dimension being measured.

### 3.4 Readability relative to a patient-facing benchmark

Responses from every platform exceeded the prespecified patient-facing reading level (**Table 4** and **Figure 2**). Six indices assessed grade-level estimates and reading ease. Because the benchmark questions were primarily specialist-facing, this analysis addresses suitability for direct patient communication rather than the quality of clinician-facing communication.

**Table 4:** AI chatbot readability scores.

Program	ChatGPT	Gemini	Copilot	Perplexity	6th-grade threshold
ARI	16.56(15.42, 17.62)	19.06(17.06, 19.93)	18.55(16.98, 18.96)	18.51(17.40, 19.59)	<6
GFI	19.21(17.92, 20.29)	20.14(19.02, 21.02)	18.90(17.94, 19.44)	18.40(17.69, 20.03)	<6
FKGL	15.93(15.19, 16.82)	16.76(15.67, 17.57)	15.87(14.69, 16.66)	16.14(14.93, 16.68)	<6
CL	21.29(20.65, 23.10)	19.52(17.16, 20.60)	21.03(19.84, 22.33)	18.39(17.75, 19.80)	<6
SMOG	13.45(13.01, 14.27)	16.91(15.97, 17.66)	14.88(14.00, 15.79)	15.90(15.47, 16.83)	<6
FRES	-4.37(-11.21, -0.15)	5.83(0.25, 12.00)	2.82(-3.70, 11.45)	11.31(9.31, 20.86)	>=80

Values are shown as median (Q1, Q3). Sixth-grade thresholds were FRES >=80 and <6 for the grade-level indices.

**Figure 2:**
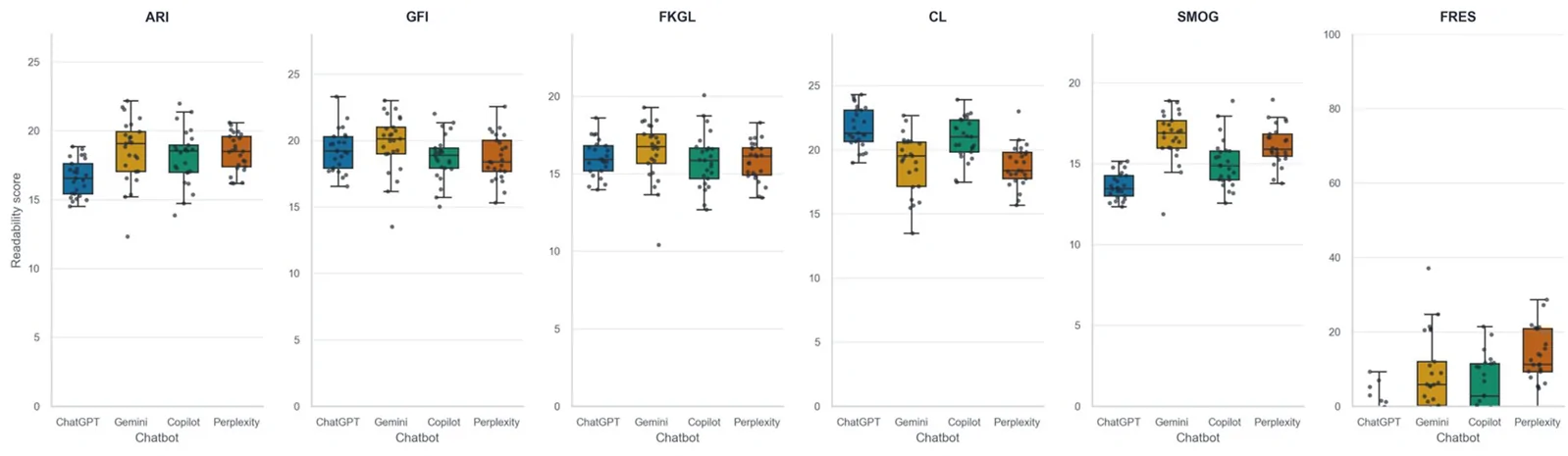
Readability of LLM-generated medical information across chatbot platforms. Boxplots show the Automated readability index (ARI), Gunning fog index (GFI), Flesch-kincaid grade level (FKGL), Coleman-liau index (CL), Simple measure of gobbledygook (SMOG), and Flesch reading ease score (FRES). Higher grade-level scores indicate greater difficulty; higher FRES values indicate easier reading. Each point represents one question-level assessment. None of the platforms reached the sixth-grade readability threshold.

All platforms exceeded the prespecified reading level for patient information. Median values ranged from 16.56 to 19.06 for ARI, 18.40 to 20.14 for GFI, 15.87 to 16.76 for FKGL, 18.39 to 21.29 for the Coleman-Liau Index, and 13.45 to 16.91 for SMOG. Each grade-level index was well above the sixth-grade threshold.

FRES values were also low, ranging from −4.37 to 11.31. Perplexity had the highest median FRES, but its responses remained far more difficult than the patient-oriented benchmark.

None of the four LLMs met the sixth-grade benchmark. Unmodified responses would therefore be difficult for many patients, although this finding does not assess their suitability for communication among clinicians.

### 3.5 Heterogeneous question-level performance patterns across LLM platforms

Question-level results varied within every platform (**Figure 3**). DISCERN, EQIP, GQS, and JAMA scores changed across benchmark items, showing that completeness, educational structure, and transparency depended on the question.

**Figure 3:**
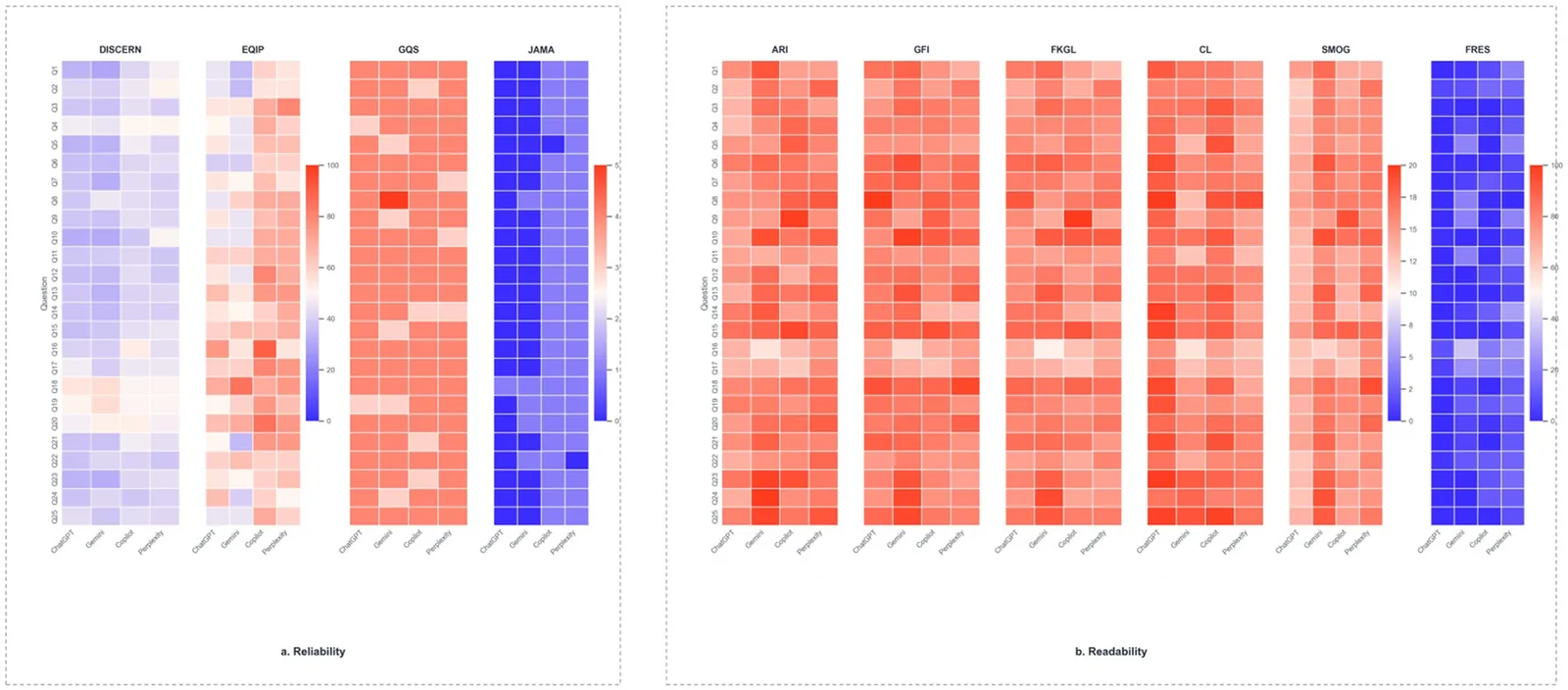
Question-level performance across LLM platforms. Heatmaps show variation across individual questions. **(A)** presents DISCERN, EQIP, Global Quality Score (GQS), and JAMA Benchmark Criteria results. **(B)** presents the Automated Readability Index (ARI), Gunning Fog Index (GFI), Flesch-Kincaid Grade Level (FKGL), Coleman-Liau Index (CL), Simple Measure of Gobbledygook (SMOG), and Flesch Reading Ease Score (FRES). Rows represent questions, columns represent chatbots, and color intensity represents relative values within each metric.

Between-platform contrasts were most visible in structured scores for questions requiring evidence appraisal or translation, including microbial toxicity signatures and evidentiary strength (Q8), barriers to biomarker translation (Q21), and integration of microbiome, clinical, and immunological data (Q24). In contrast, GQS ratings were largely similar across questions, including many items on core mechanisms and differential assessment.

Readability was more consistent: most responses to most questions exceeded recommended patient-education reading levels, despite variation across platforms. Translating specialized evidence into accessible language remained a shared weakness.

The observed variation was task- and metric-dependent and did not establish differences in clinical reasoning accuracy. Poor patient-facing readability persisted across the benchmark.

## 4 Discussion

This disease-specific benchmark demonstrates that current LLM-based chatbots exhibit heterogeneous performance on ICI-LI clinical information synthesis tasks with a focused microbiome component. Although all evaluated models generated structured medical responses, their performance varied across information quality, transparency, and readability relative to a patient-facing benchmark. Notably, different evaluation instruments captured distinct aspects of model performance, indicating that medical AI systems should not be assessed through a single global measure (Wu et al., 2025; Zhou et al., 2025). These findings support multidimensional evaluation of information quality and communication, but they do not establish clinical reasoning accuracy or biomedical competence.

ICI-LI represents a challenging setting for clinical AI evaluation because diagnosis and management require integration of complex biological and clinical information (Triantafyllou et al., 2025). Unlike conventional drug-induced liver injury, ICI-LI encompasses heterogeneous phenotypes, including hepatocellular injury, cholangitis-like manifestations, and immune-mediated inflammatory patterns, while requiring differentiation from competing causes of hepatic dysfunction (Haanen et al., 2022). Microbiome findings add a specific calibration problem because their biological plausibility exceeds their current clinical actionability (Gopalakrishnan et al., 2018). Differences in cancer populations, treatment regimens, sequencing strategies, and analytical approaches further complicate interpretation of microbiome findings (McCulloch et al., 2022). Most available microbiome data concern ICI response or irAEs across cancer types, whereas direct evidence for ICI-LI remains sparse. LLM responses should therefore distinguish biological plausibility, associative evidence, and clinically actionable evidence. In an emerging biomarker field, evidence calibration may be more clinically important than information volume.

The instruments differentiated platforms because they emphasize different response features. DISCERN and EQIP assess specified elements of content and structure, whereas JAMA assesses disclosure attributes. GQS provides a global impression and may compress differences in completeness, evidence attribution, and transparency. Its limited discrimination in this study supports complementary rather than isolated use of global ratings (Alderman et al., 2025; Blom and Pani, 2026). Higher JAMA scores may also reflect platform-level citation or source-display functions rather than superior biomedical competence. The JAMA Benchmark Criteria were originally developed for static online health resources and may not fully capture the characteristics of generative AI outputs (Agrawal et al., 2025). These instruments should therefore be interpreted as complementary indicators, and future AI-specific measures should assess factual accuracy, evidence traceability, and uncertainty communication directly.

The readability findings address a different task from specialist information synthesis. Complex terminology and high linguistic demands limit direct patient-facing use of unmodified responses (Ayers et al., 2023). Specialist responses may remain appropriate for clinicians despite high grade-level scores, whereas patient explanations of toxicity, monitoring, and treatment choices require deliberate adaptation. Patient-oriented systems should preserve medical accuracy while adjusting language complexity to health literacy (Raghu Subramanian et al., 2024; Mahajan and Powell, 2025).

Potential clinical uses should be defined by task risk. LLMs may assist with organizing literature, summarizing established guidance, and preparing clinician-reviewed explanations (Singhal et al., 2023). They should not independently interpret microbiome biomarkers for an individual patient, determine immunosuppressive treatment, or recommend ICI rechallenge. These higher-risk tasks require verified evidence and clinician judgment (van de Sande et al., 2024; Curcin et al., 2026). Future studies should test factual accuracy, calibration to evidence maturity, and the effect of LLM output on task-specific clinical decisions.

Several limitations should be acknowledged. First, only four publicly available platforms were evaluated at one time point, and subsequent updates may change performance. Second, the benchmark addressed one disease setting and included both general ICI-LI questions and a focused microbiome component, which limits generalizability. Third, the study assessed information quality, structure, transparency, and readability but not factual accuracy, diagnostic performance, or effects on clinical decisions. Fourth, DISCERN, EQIP, GQS, and the JAMA Benchmark Criteria were developed for consumer-oriented or web-based health information rather than generative responses to specialist biomedical questions. Their scores are complementary indicators and should not be interpreted as direct measures of medical competence. Finally, the sixth-grade threshold evaluates patient-facing accessibility; it does not define the appropriate complexity of clinician-facing communication. Studies involving clinicians and patients are needed to evaluate task-specific utility.

## 5 Conclusions

Current LLM chatbots differ in the quality, structure, and transparency of information generated for ICI-LI, and unmodified responses did not meet the patient-facing readability benchmark. The microbiome component showed the importance of calibrating claims to evidence maturity. Disease-specific multidimensional evaluation is needed before LLM-generated information can be considered reliable for clinical communication or decision support, particularly for biomarker interpretation and treatment decisions.

## Data Availability

The original contributions presented in the study are included in the article/**Supplementary Material**. Further inquiries can be directed to the corresponding author.

## References

[B1] Agrawal M. Chen I. Y. Gulamali F. Joshi S. (2025). The evaluation illusion of large language models in medicine. NPJ Digital Med. 8, 600. doi: 10.1038/s41746-025-01963-x 41057566 PMC12504413

[B2] Albillos A. de Gottardi A. Rescigno M. (2020). The gut-liver axis in liver disease: pathophysiological basis for therapy. J. Hepatol. 72, 558–577. doi: 10.1016/j.jhep.2019.10.003 31622696

[B3] Alderman J. E. Palmer J. Laws E. McCradden M. D. Ordish J. Ghassemi M. . (2025). Tackling algorithmic bias and promoting transparency in health datasets: the STANDING together consensus recommendations. Lancet DIGIT. Health 7, e64–e88. doi: 10.1016/S2589-7500(24)00224-3 39701919 PMC11668905

[B4] Andrews M. C. Duong C. P. M. Gopalakrishnan V. Iebba V. Chen W.-S. Derosa L. . (2021). Gut microbiota signatures are associated with toxicity to combined CTLA-4 and PD-1 blockade. Nat. Med. 27, 1432–1441. doi: 10.1038/s41591-021-01406-6 34239137 PMC11107795

[B5] Ayers J. W. Poliak A. Dredze M. Leas E. C. Zhu Z. Kelley J. B. . (2023). Comparing physician and artificial intelligence chatbot responses to patient questions posted to a public social media forum. JAMA Intern. Med. 183, 589–596. doi: 10.1001/jamainternmed.2023.1838 37115527 PMC10148230

[B6] Berkman N. D. Sheridan S. L. Donahue K. E. Halpern D. J. Crotty K. (2011). Low health literacy and health outcomes: an updated systematic review. Ann. Intern. Med. 155, 97–107. doi: 10.7326/0003-4819-155-2-201107190-00005 21768583

[B7] Bernard A. Langille M. Hughes S. Rose C. Leddin D. Veldhuyzen van Zanten S. (2007). A systematic review of patient inflammatory bowel disease information resources on the world wide web. Am. J. Gastroenterol. 102, 2071–2077. doi: 10.1111/j.1572-0241.2007.01325.x 17511753

[B8] Berry P. Kotha S. Zen Y. Papa S. El Menabawey T. Webster G. . (2023). Immune checkpoint inhibitor-related cholangiopathy: novel clinicopathological description of a multi-centre cohort. Liver Int. Off. J. Int. Assoc. Study Liver 43, 147–154. doi: 10.1111/liv.15340 35704341

[B9] Blom J. M. C. Pani L. (2026). Embedded transparency in artificial intelligence: a prerequisite for equity and representation in AI-enabled clinical trials. NPJ Digital Med. 9, 531. doi: 10.1038/s41746-026-02987-7 42436283 PMC13356023

[B10] Brahmer J. R. Lacchetti C. Schneider B. J. Atkins M. B. Brassil K. J. Caterino J. M. . (2018). Management of immune-related adverse events in patients treated with immune checkpoint inhibitor therapy: american society of clinical oncology clinical practice guideline. J. Clin. Oncol. Off. J. Am. Soc Clin. Oncol. 36, 1714–1768. doi: 10.1200/JCO.2017.77.6385 29442540 PMC6481621

[B11] Charnock D. Shepperd S. Needham G. Gann R. (1999). DISCERN: an instrument for judging the quality of written consumer health information on treatment choices. J. Epidemiol. Community Health 53, 105–111. doi: 10.1136/jech.53.2.105 10396471 PMC1756830

[B12] Curcin V. Delaney B. Alkhatib A. Cockburn N. Dann O. Kostopoulou O. . (2026). Learning health systems provide a glide path to safe landing for AI in health. Artif. Intell. Med. 173, 103346. doi: 10.1016/j.artmed.2025.103346 41506062

[B13] Dougan M. Wang Y. Rubio-Tapia A. Lim J. K. (2021). AGA clinical practice update on diagnosis and management of immune checkpoint inhibitor colitis and hepatitis: expert review. Gastroenterology 160, 1384–1393. doi: 10.1053/j.gastro.2020.08.063 33080231

[B14] Gkikas D. C. Tzafilkou K. Theodoridis P. K. Garmpis A. Gkikas M. C. (2022). How do text characteristics impact user engagement in social media posts: modeling content readability, length, and hashtags number in Facebook. Int. J. Inf. Manage. Data Insights 2, 100067. doi: 10.1016/j.jjimei.2022.10006742574925

[B15] Gopalakrishnan V. Spencer C. N. Nezi L. Reuben A. Andrews M. C. Karpinets T. V. . (2018). Gut microbiome modulates response to anti-PD-1 immunotherapy in melanoma patients. Science 359, 97–103. doi: 10.1126/science.aan4236 29097493 PMC5827966

[B16] Haanen J. Obeid M. Spain L. Carbonnel F. Wang Y. Robert C. . (2022). Management of toxicities from immunotherapy: ESMO clinical practice guideline for diagnosis, treatment and follow-up. Ann. Oncol. Off. J. Eur. Soc Med. Oncol. 33, 1217–1238. doi: 10.1016/j.annonc.2022.10.001 36270461

[B17] Hanci V. Otlu B. Biyikoğlu A. S. (2024). Assessment of the readability of the online patient education materials of intensive and critical care societies. Crit. Care Med. 52, e47–e57. doi: 10.1097/CCM.0000000000006121 37962133

[B18] Kung T. H. Cheatham M. Medenilla A. Sillos C. De Leon L. Elepaño C. . (2023). Performance of ChatGPT on USMLE: potential for AI-assisted medical education using large language models. PLoS Digital Health 2, e0000198. doi: 10.1371/journal.pdig.0000198 36812645 PMC9931230

[B19] Lee P. Bubeck S. Petro J. (2023). Benefits, limits, and risks of GPT-4 as an AI chatbot for medicine. N. Engl. J. Med. 388, 1233–1239. doi: 10.1056/NEJMsr2214184 36988602

[B20] Lee K. A. Thomas A. M. Bolte L. A. Björk J. R. de Ruijter L. K. Armanini F. . (2022). Cross-cohort gut microbiome associations with immune checkpoint inhibitor response in advanced melanoma. Nat. Med. 28, 535–544. doi: 10.1038/s41591-022-01695-5 35228751 PMC8938272

[B21] Mac O. Ayre J. Bell K. McCaffery K. Muscat D. M. (2022). Comparison of readability scores for written health information across formulas using automated vs manual measures. JAMA Netw. Open 5, e2246051. doi: 10.1001/jamanetworkopen.2022.46051 36508219 PMC9856555

[B22] Mahajan A. Powell D. (2025). Transforming healthcare delivery with conversational AI platforms. NPJ Digital Med. 8, 581. doi: 10.1038/s41746-025-01968-6 41028209 PMC12484644

[B23] Matson V. Fessler J. Bao R. Chongsuwat T. Zha Y. Alegre M.-L. . (2018). The commensal microbiome is associated with anti-PD-1 efficacy in metastatic melanoma patients. Science 359, 104–108. doi: 10.1126/science.aao3290 29302014 PMC6707353

[B24] McCulloch J. A. Davar D. Rodrigues R. R. Badger J. H. Fang J. R. Cole A. M. . (2022). Intestinal microbiota signatures of clinical response and immune-related adverse events in melanoma patients treated with anti-PD-1. Nat. Med. 28, 545–556. doi: 10.1038/s41591-022-01698-2 35228752 PMC10246505

[B25] Moult B. Franck L. S. Brady H. (2004). Ensuring quality information for patients: development and preliminary validation of a new instrument to improve the quality of written health care information. Health Expect.: Int. J. Public Particip. Health Care Health Policy 7, 165–175. doi: 10.1111/j.1369-7625.2004.00273.x 15117391 PMC5060233

[B26] Ojha P. K. Ismail A. Kundumani K. Srinivasan (2021). Perusal of readability with focus on web content understandability. J. King Saud Univ. Comput. Inf. Sci. 33, 1–10. doi: 10.1016/j.jksuci.2018.03.00742574925

[B27] Özduran E. Hanci V. (2022). Evaluating the readability, quality and reliability of online information on behçet’s disease. Reumatismo 74, 49–60. doi: 10.4081/reumatismo.2022.1495 36101989

[B28] Postow M. A. Sidlow R. Hellmann M. D. (2018). Immune-related adverse events associated with immune checkpoint blockade. N. Engl. J. Med. 378, 158–168. doi: 10.1056/NEJMra1703481 29320654

[B29] Raghu Subramanian C. Yang D. A. Khanna R. (2024). Enhancing health care communication with large language models-the role, challenges, and future directions. JAMA Netw. Open 7, e240347. doi: 10.1001/jamanetworkopen.2024.0347 38466311

[B30] Regev A. Avigan M. I. Kiazand A. Vierling J. M. Lewis J. H. Omokaro S. O. . (2020). Best practices for detection, assessment and management of suspected immune-mediated liver injury caused by immune checkpoint inhibitors during drug development. J. Autoimmun. 114, 102514. doi: 10.1016/j.jaut.2020.102514 32768244

[B31] Ribas A. Wolchok J. D. (2018). Cancer immunotherapy using checkpoint blockade. Science 359, 1351–1355. doi: 10.1126/science.aar4060 29567705 PMC7391259

[B32] Silberg W. M. Lundberg G. D. Musacchio R. A. (1997). Assessing, controlling, and assuring the quality of medical information on the internet: caveant lector et viewor--let the reader and viewer beware. JAMA 277, 1244–1245. doi: 10.1001/jama.1997.03540390074039 9103351

[B33] Singhal K. Azizi S. Tu T. Mahdavi S. S. Wei J. Chung H. W. . (2023). Large language models encode clinical knowledge. Nature 620, 172–180. doi: 10.1038/s41586-023-06291-2 37438534 PMC10396962

[B34] Triantafyllou E. Gudd C. L. C. Possamai L. A. (2025). Immune-mediated liver injury from checkpoint inhibitors: mechanisms, clinical characteristics and management. Nat. Rev. Gastroenterol. Hepatol. 22, 112–126. doi: 10.1038/s41575-024-01019-7 39663461

[B35] Tripathi A. Debelius J. Brenner D. A. Karin M. Loomba R. Schnabl B. . (2018). The gut-liver axis and the intersection with the microbiome. Nat. Rev. Gastroenterol. Hepatol. 15, 397–411. doi: 10.1038/s41575-018-0011-z 29748586 PMC6319369

[B36] van de Sande D. Chung E. F. F. Oosterhoff J. van Bommel J. Gommers D. van Genderen M. E. (2024). To warrant clinical adoption AI models require a multi-faceted implementation evaluation. NPJ Digital Med. 7, 58. doi: 10.1038/s41746-024-01064-1 38448743 PMC10918103

[B37] Wu C. Qiu P. Liu J. Gu H. Li N. Zhang Y. . (2025). Towards evaluating and building versatile large language models for medicine. NPJ Digital Med. 8, 58. doi: 10.1038/s41746-024-01390-4 39865143 PMC11770143

[B38] Yilmaz Hanci S. (2023). How readable and quality are online patient education materials about helicobacter pylori?: assessment of the readability, quality and reliability. Med. (Baltimore) 102, e35543. doi: 10.1097/MD.0000000000035543 37904459 PMC10615431

[B39] Zhou S. Xie W. Li J. Zhan Z. Song M. Yang H. . (2025). Automating expert-level medical reasoning evaluation of large language models. NPJ Digital Med. 9, 34. doi: 10.1038/s41746-025-02208-7 41353516 PMC12796170

