# Supplementary material for: Evaluation of large language models for ICI-related liver injury with an emerging microbiome focus
Source: Front Cell Infect Microbiol. 2026 Oct 9;16:1955356. doi: 10.3389/fcimb.2026.1955356 (PMC13690555; doi:10.3389/fcimb.2026.1955356)
Supplement: Supplementary file 1 [file Table2.docx]

Supplementary Table 2. Scoring criteria and interpretation of health information quality and accessibility assessment tools

| **Tool** | **Purpose** | **Scoring method** | **Interpretation** |
| --- | --- | --- | --- |
| DISCERN | Evaluates the quality, reliability, transparency, and balance of health information | 16-item instrument scored from 1 to 5 per item, with total scores ranging from 16 to 75 | 63–75: excellent; 51–62: good; 39–50: fair; 27–38: poor; 16–26: very poor |
| EQIP | Evaluates the quality, content completeness, presentation, and usability of patient education materials | 20-item checklist. Each item scored as “Yes” = 1 or “No” = 0. Final score calculated as (total score/20) ×100 | 76–100: excellent; 51–75: good; 26–50: poor; 0–25: serious quality issues |
| GQS | Evaluates overall educational quality, organization, and usefulness for patients | Five-point Likert scale | 5: excellent; 4: good; 3: fair; 2: poor; 1: very poor |
| JAMA benchmark | Evaluates transparency and reliability indicators including authorship, attribution, disclosure, and currency | Four criteria scored as 0 or 1, with total scores ranging from 0 to 4 | Higher scores indicate greater transparency |
