# Supplementary material for: Evaluation of large language models for ICI-related liver injury with an emerging microbiome focus
Source: Front Cell Infect Microbiol. 2026 Oct 9;16:1955356. doi: 10.3389/fcimb.2026.1955356 (PMC13690555; doi:10.3389/fcimb.2026.1955356)
Supplement: Supplementary file 2 [file Table3.docx]

Supplementary Table 3. Readability indices used to assess accessibility of AI-generated cancer information

| **Readability index** | **Purpose** | **Formula** | **Interpretation** |
| --- | --- | --- | --- |
| ARI | Estimates US grade level based on characters and word structure | Formula | Higher scores indicate greater complexity |
| GFI | Estimates years of education required | Formula | Higher scores indicate greater complexity |
| FKGL | Estimates grade level required | Formula | Higher scores indicate greater complexity |
| CLI | Estimates grade level based on sentence and character features | Formula | Higher scores indicate greater complexity |
| SMOG | Estimates education level based on polysyllabic words | Formula | Higher scores indicate greater complexity |
| FRES | Measures reading ease | Formula | Higher scores indicate better readability |
